# Impaired Systolic and Diastolic Left Ventricular Function in Children with Chronic Kidney Disease - Results from the 4C Study

**DOI:** 10.1038/s41598-019-46653-3

**Published:** 2019-08-07

**Authors:** Anke Doyon, Pascal Haas, Sevcan Erdem, Bruno Ranchin, Behrouz Kassai, Francesca Mencarelli, Francesca Lugani, Jerome Harambat, Maria Chiara Matteucci, Marcello Chinali, Sandra Habbig, Ariane Zaloszyc, Sara Testa, Enrico Vidal, Charlotte Gimpel, Karolis Azukaitis, Alexander Kovacevic, Uwe Querfeld, Franz Schaefer

**Affiliations:** 1 Division of Pediatric Nephrology, Center for Pediatrics and Adolescent Medicine, Heidelberg, Germany; 2 Division of Pediatric Cardiology, Cukurova University, Adana, Turkey; 3 Department of Pediatric Nephrology, Rheumatology and Dermatology, Hospices Civils de Lyon, Lyon, France; 4 Service de Pharmacotoxicologie, Centre d’Investigation Clinique, 1407 Inserm, UMR 5558, LBBE, CNRS Lyon, Université de Lyon and Hospices Civils de Lyon, Lyon, France; 5 Nephrology and Dialysis Unit, Department of Pediatrics, Azienda Ospedaliero Universitaria Sant Orsola-Malpighi, Bologna, Italy; 6 Department of Pediatric Nephrology, Istituto Giannina Gaslini, Genova, Italy; 7 Pediatric Nephrology Unit, Department of Pediatrics, Bordeaux University Hospital, Bordeaux, France; 8 Department of Nephrology and Urology, Bambino Gesù Pediatric Hospital, Rome, Italy; 9 Department of Cardiology, Bambino Gesù Pediatric Hospital, Rome, Italy; 10 University Childrens’ and Adolescents’ Hospital Cologne, Cologne, Germany; 11 Pediatric Nephrology Unit, Hautepierre University Hospital, Strasbourg, France; 12 Pediatric Nephrology, Dialysis and Transplant Unit, Fondazione Osp Maggiore Policlinico, Milan, Italy; 13 Pediatric Nephrology, Dialysis and Transplant Unit, Department of Womens and Childrens Health, University Hospital of Padova, Padua, Italy; 14 Department of General Pediatrics, Adolescent Medicine and Neonatology, Center for Pediatrics, Medical Center – University of Freiburg, Faculty of Medicine, Freiburg im Breisgau, Germany; 15 Center for Pediatrics, Vilnius University, Vilnius, Lithuania; 16 Department of Pediatric and Congenital Cardiology, Center for Pediatrics and Adolescent Medicine, Heidelberg, Germany; 17 Division of Pediatric Nephrology, Charite Children’s Hospital, Berlin, Germany; 18 4C Study Consortium, Heidelberg, Germany

**Keywords:** Hypertension, Atherosclerosis, Carotid artery disease, Chronic kidney disease

## Abstract

Children with chronic kidney disease suffer from excessive cardiovascular mortality and early alterations of the cardiovascular system. Tissue doppler imaging is a validated echocardiographic tool to assess early systolic and diastolic cardiac dysfunction. We hypothesized that tissue Doppler velocities would reveal reduced cardiac function in children with chronic kidney disease compared to healthy children. A standardized echocardiographic exam was performed in 128 patients of the Cardiovascular Comorbidity in Children with Chronic Kidney Disease (4C) Study aged 6–17 years with an estimated glomerular filtration rate (eGFR) below 60 ml/min/1.73 m^2^. Tissue Doppler measurements included early (E’) and late (A’) diastolic and systolic (S’) velocity at the mitral and septal annulus of the left ventricle. Measured values were normalized to z-scores using published reference data. Predictors of E’/A’, E/E’, S’ and left ventricular mass index (LVMI) were assessed by multiple linear regression analyses. Tissue Doppler E’ was reduced and tissue Doppler A’ increased, resulting in a reduced tissue Doppler E’/A’ ratio (z-score −0.14, p < 0.0001) indicating reduced diastolic function compared to healthy children. Reduced tissue Doppler E’/A’ Z-Scores were independently associated with lower eGFR (p = 0.002) and increased systolic blood pressure (p = 0.02). While E/E’ Z-Scores were increased (Z-score 0.57, p < 0.0001), patients treated with pharmacological RAS blockade but not with other antihypertensive treatments had significantly lower E/E’ and higher E’/A’ Z-Scores. Systolic tissue Doppler velocities were significantly decreased (Z-score −0.24, p = 0.001) and inversely correlated with E/E’ Z-Scores (r = −0.41, p < 0.0001). LVMI was not associated with systolic or diastolic tissue Doppler velocities. Concentric left ventricular hypertrophy showed a tendency to lower S’ in multivariate analysis (p = 0.13) but no association to diastolic function. Concentric left ventricular geometry was significantly associated with lower midwall fractional shortening. In summary, systolic and diastolic function assessed by tissue Doppler is impaired. eGFR, systolic blood pressure and the type of antihypertensive medications are significant predictors of diastolic function in children with CKD. Left ventricular morphology is largely independent of tissue Doppler velocities. Tissue Doppler velocities provide sensitive information about early left ventricular dysfunction in this population.

## Introduction

Chronic kidney disease (CKD) is associated with type 4 cardiorenal syndrome and an excessive mortality from cardiovascular disease (CVD). The rate of fatal cardiac events is highly increased in children on dialysis or after kidney transplantation compared to the general population and is the leading cause of death in this patient population^1^. Children with CKD are the population with the highest risk for cardiovascular disease. Alterations of the cardiovascular system can be identified at an early age in pediatric populations^1,2^.

Non-invasive exercise tests have shown impaired cardiac performance indices in asymptomatic adult pre-dialysis CKD patients^3^. Likewise in children with CKD, subclinical systolic dysfunction has been described using sophisticated echocardiographic measurements such as strain analysis and endocardial and midwall fractional shortening, while ejection fraction is usually normal^4,5^. These findings add to conventional echocardiographic studies with distinct changes in cardiac morphology and geometry in children with CKD^4–7^. Tissue Doppler measurements are a reasonable echocardiographic tool in pediatrics since they are non-invasive, hardly time-consuming, widely available and software independent compared to other measurements. The measurement of diastolic indices by Tissue doppler is of particular interest since diastolic dysfunction is regarded as the main abnormality in heart failure with preserved ejection fraction and in adults it is predictive of progress to overt heart failure^8^. Diastolic dysfunction has also been described as an integral part of cardiorenal syndrome type 4, which is increasingly diagnosed in adults with CKD and is associated with increased mortality rates up to an odds ratio of 4.4 for an eGFR of 40 ml/min/1.73 m^2^ ^9,10^. Accordingly, the use of tissue Doppler imaging for evaluation of both diastolic and systolic function has been recommended by the American Society of Echocardiography and the European Society of Cardiology^11,12^.

Tissue Doppler measurements include both diastolic and systolic measurements (early diastolic mitral annulus velocity E’, late diastolic velocity A’, systolic velocity S’). Derived measures are the E’/A’ ratio describing diastolic function and conventional E to tissue Doppler E’ ratio (E/E’ ratio), a surrogate of left ventricular filling pressure.

Few tissue Doppler studies, in small cohorts, have been conducted to date to evaluate both myocardial diastolic or systolic function in pediatric kidney disease^13–16^. Since pediatric reference values have been provided, quantification of abnormal states across the pediatric age range is readily possible^17^.

The present study aimed to describe diastolic and systolic function by tissue Doppler parameters in a large cohort of children with CKD and to identify factors associated with impaired systolic or diastolic function.

## Results

### Subject characteristics and conventional echocardiographic parameters

The characteristics of the 128 patients are shown in Table 1. BP was elevated and increased with CKD stage. Serum parameters for albumin, hemoglobin and bicarbonate were stable across CKD stages, whereas serum inorganic phosphorus and iPTH levels increased with CKD stage. 61% of all patients were on antihypertensive medication; 44.5% received RAS inhibitors. 19.5% of patients were treated with combined antihypertensive medications, including a RAS inhibitor in 11.7%. For further description of antihypertensive medication, see Table 1.

**Table 1:** Subject characteristics in 128 children with CKD. Findings are stratified by CKD stage.

N	All	CKD 2-3b	CKD 4–5	Dialysis
	128	46	69	13
Age (years)	12.7 ± 3.5	12.8 ± 3.25	12.4	14.3
% male	72.7	69.6	73.9	76.9
BMI z-score	0.16 ± 1.10	0.13 ± 1.22	0.19 ± 1.08	0.07 ± 0.83
Height z-score	−0.98 ± 1.08	−0.83 ± 0.91	−0.96 ± 1.08	−1.66 ± 1.44
Systolic BP z-score	0.59 ± 1.14	0.46 ± 1.11	0.54 ± 1.10	1.33 ± 1.26
Diastolic BP z-score	0.31 ± 0.97	0.28 ± 0.83	0.3 ± 1.03	0.41 ± 1.16
eGFR (ml/min/1.73 m^2^)	25.8 ± 11.4	38.1 ± 6.83	21 ± 4.55	n.a.
Serum albumin (g/L)	39.9 ± 3.67	39.7 ± 3.71	40.3 ± 3.47	37.6 ± 4.12
Serum hemoglobin (g/dl)	11.9 ± 1.77	12.3 ± 2.3	11.8 ± 1.35	11.09 ± 1.37
Serum LDL cholesterol (mg/dl)	106 ± 36	105 ± 35	110 ± 37	85 ± 23
Serum HDL cholesterol (mg/dl)	53.6 ± 13.8	56.4 ± 15.0	52.0 ± 13.1	51.4 ± 11.6
Serum bicarbonate (mM)	22.9 ± 3.66	23.6 ± 2.78	21.9 ± 3.75	25.6 ± 4.5
Serum calcium (mM)	2.35 ± 0.18	2.33 ± 0.15	2.34 ± 0.20	2.46 ± 0.18
Serum phosphate (mM)	1.53 ± 0.29	1.40 ± 0.21	1.59 ± 0.31	1.69 ± 0.29
Serum iPTH (pmol/l)	13.7 (19.5)	11.6 (32.7)	15.3 (17.1)	17.0 (18.4)
CRP (mg/l)	0.39 (0.9)	0.3 (0.91)	0.37 (0.62)	0.7 (1.57)
Albuminuria (mg/g creatinine)	288 (849)	177 (452)	324 (967)	618 (1197)
**Antihypertensive medication (n**, **%)**	78 (60.9)	25 (54.3)	45 (65.2)	8 (61.5)
RAS Inhibitor	57 (44.5)	21 (45.7)	31 (44.9)	5 (38.5)
Calcium blockers	25 (19.5)	6 (13)	18 (26.1)	1 (7.7)
Diuretics	9 (7)	1 (2.2)	5 (7.3)	3 (23.1)
Other BP medication	18 (14.1)	1 (2.2)	13 (18.8)	4 (30.8)
*Mono- or combination therapy*				
RAS Inhibitor monotherapy	42 (32.8)	18 (39.1)	20 (29.0)	4 (30.8)
RAS Inhibitor + other AHT	15 (11.7)	3 (6.5)	11 (15.9)	1 (7.7)
Non-RAS inhibitor monotherapy	11 (8.6)	3 (6.5)	7 (10.1)	1 (7.7)
Non-RAS combination therapy	10 (7.8)	1 (2.2)	7 (10.1)	2 (15.4)
**Other medications**				
Vitamin D supplement	32 (25)	9 (19.6)	21 (30.4)	2 (15.4)
Calcitriol	74 (57.8)	20 (43.5)	46 (66.7)	8 (61.5)
Phosphate binders	54 (42.2)	11 (23.9)	30 (43.5)	13 (100)
Erythropoiesis stimulating agent	54 (42.2)	12 (26.1)	31 (44.9)	11 (84.6)

Values are Mean ± SD or Median (IQR) as appropriate; eGFR, estimated Glomerular Filtration Rate; BP, Blood Pressure; RAS, Renin Angiotensin System; AHT, Antihypertensive Therapy.

### Diastolic function by conventional and tissue Doppler

**Figure 1:**
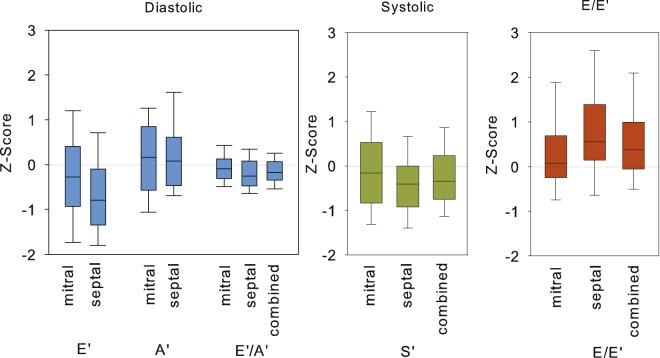
Distribution of left ventricular tissue Doppler measures in 128 children with CKD. Data are expressed as Z-scores. (LVFP = Left ventricular filling pressure, boxes and whiskers: Median, 10^th^, 25^th^, 75^th^ and 90^th^ percentiles).

Diastolic function measured by conventional Doppler (E and E/A) ratio over the mitral valve was not different among CKD groups (Table 2). Tissue Doppler results are shown in Table 3 (TD Z-Scores), Table S1 (Unadjusted TD velocities) and Fig. 1 (TD Z-Scores). All correlation and regression analysis were performed with tissue Doppler Z-Scores. While diastolic TD velocities of the patients were mostly within normal ranges, Z-Scores of the early diastolic TD velocity (E’) were reduced significantly while the late diastolic TD velocity (A’) was significantly increased compared to a previously published healthy population^17^ (Table 3, Fig. 1). This resulted in a decrease of the TD E’/A’ ratio Z-Scores, suggesting reduced diastolic function compared to healthy controls. The reduction of TD E’/A’ Z-Scores were more pronounced at the septum. Diastolic TD velocities E’ and A’ Z-Scores were highly correlated to each other and to systolic TD velocity Z-Score (S’) and E/E’ Z-Scores (Table S2, Supplement). Univariate analysis revealed significantly decreased TD E’/A’ Z-Scores in dialysis patients and patients with CKD stage 4–5 compared to patients with milder chronic kidney disease (p < 0.05). In multivariate analysis, reduced TD E’/A’ Z-Scores were associated with lower eGFR and increased systolic blood pressure. Patients treated with RAS inhibitors had a higher TD E’/A’ Z-Score ratio compared to untreated patients (TD E’/A’ −0.07 ± 0.31 vs −0.20 ± 0.29, p = 0.02) in univariate and multivariate analysis.

**Table 2:** Conventional echocardiographic findings in 128 children with CKD, stratified by CKD stage.

N	All	CKD 2-3b	CKD 4-5	Dialysis
	128	46	69	13
LVMI (g/m^2.16^ + 0.09)	45.2 ± 10.4	41.2 ± 8.15	46.5 ± 10.5	50.6 ± 13.5
Relative Wall Thickness	0.43 ± 0.25	0.42 ± 0.11	0.43 ± 0.13	0.49 ± 0.20
LV hypertrophy	60 (46.9%)	16 (34.8%)	35 (50.7%)	9 (69.2%)
Eccentric	20 (15.6%)	5 (10.9%)	22 (20.3%)	1 (7.7%)
Concentric	40 (31.2%)	22 (23.9%)	14 (30.4%)	8 (61.5%)
Concentric LV remodeling	38 (29.7%)	16 (34.8%)	22 (31.9%)	—
Endocardial fractional shortening (EFS)	37.9 ± 7.72	39.5 ± 8.19	36.3 ± 7.49	40.6 ± 5.60
Midwall fractional shortening (MFS)	16.4 ± 3.58	17.3 ± 3.48	15.8 ± 3.58	15.9 ± 3.54
E	99.3 ± 16.8	98.2 ± 14.6	99.4 ± 1.85	103.1 ± 17.15
E/A Ratio	1.89 ± 0.57	1.94 ± 0.55	1.86 ± 0.61	1.87 ± 0.46
IVS diastolic (cm)	0.90 ± 0.19	0.86 ± 0.16	0.91 ± 0.18	0.96 ± 0.26
LVID diastolic (cm)	3.95 ± 0.56	3.95 ± 0.54	3.95 ± 0.57	3.99 ± 0.63
PW diastolic (cm)	0.86 ± 0.19	0.84 ± 0.16	0.86 ± 0.19	0.98 ± 0.25
IVS systolic (cm)	1.4 ± 1.27	1.59 ± 2.06	1.26 ± 0.27	1.45 ± 0.24

LVMI, Left ventricular mass index; E, Early diastolic velocity (cm/s); A, Atrial; E/A ratio Early to atrial diastolic Doppler flow velocity; IVS Interventricular septum (cm); LVID Left ventricular internal diameter (cm); PW Posterior wall (cm).

**Table 3:** Tissue Doppler Results in 128 children with CKD, stratified by CKD stage. Tissue Doppler Velocities (Z-Scores, Mean ± SD).

	All	CKD 2-3b	CKD 4-5	Dialysis
**E’**				
Mitral anular	−0.24 ± 1.07*	−0.14 ± 1.20	−0.24 ± 0.99	−0.62 ± 0.94
Septal	−0.69 ± 1.02***	−0.60 ± 1.10	−0.77 ± 1.00	−0.58 ± 0.88
Combined	−0.45 ± 0.94***	−0.37 ± 1.05	−0.48 ± 0.90	−0.60 ± 0.77
**A’**				
Mitral anular	0.21 ± 1.11*	−0.02 ± 0.97^B;C^	0.21 ± 1.11^A^	1.01 ± 1.35^A^
Septal	0.22 ± 0.96***	0.04 ± 1.02	0.33 ± 0.95	0.29 ± 0.74
Combined	0.23 ± 0.95*	0.01 ± 0.90^B^	0.30 ± 0.96^A^	0.65 ± 0.89
**S’**				
Mitral anular	−0.11 ± 1.03	−0.17 ± 1.05	−0.10 ± 1.01	0.09 ± 1.10
Septal	−0.38 ± 0.93***	−0.39 ± 0.81	−0.43 ± 1.03	−0.09 ± 0.81
Combined	−0.24 ± 0.85*	−0.28 ± 0.84	−0.26 ± 0.86	0.002 ± 0.90
**E’/A’ (diastolic function)**				
Mitral anular	−0.08 ± 0.34*	0.03 ± 0.35^C^	−0.09 ± 0.31^C^	−0.40 ± 0.18^B^
Septal	−0.20 ± 0.40***	−0.10 ± 0.43	−0.26 ± 0.36	−0.19 ± 0.40
Combined	−0.14 ± 0.3***	−0.03 ± 0.34^B,C^	−0.18 ± 0.28^A^	−0.29 ± 0.18 ^A^
**E/E’ (LV compliance)**				
Mitral anular	0.34 ± 0.98**	0.18 ± 0.83	0.35 ± 1.01	0.82 ± 1.18
Septal	0.81 ± 1.21***	0.63 ± 1.10	0.95 ± 1.22	0.85 ± 1.54
Combined	0.57 ± 1.00***	0.40 ± 0.89	0.64 ± 1.01	0.83 ± 1.31

*p < 0.05;**p < 0.001;***p < 0.0001 compared to reference population.

A: different from CKD 2-3b; B: different from CKD 4–5; C: different from Dialysis (all p < 0.05).

**Figure 2:**
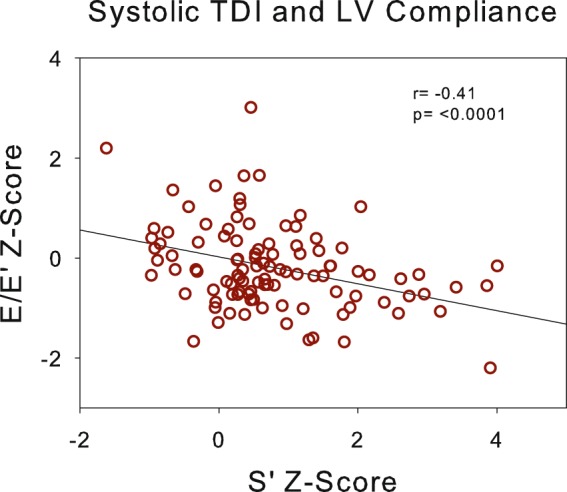
Association of left ventricular filling pressure (E/E’) with systolic tissue Doppler Velocity (S’).

E/E’ Z-Score, a surrogate for left ventricular filling pressure was significantly elevated both at the mitral and septal annulus (Table 3, Fig. 1). E/E’ Z-Score was highly negatively correlated to systolic TD velocity Z-Score (Fig. 2). While E/E’ Z-Score was not associated to either eGFR or systolic BP SDS, patients treated with RAS inhibitors had significantly lower E/E’ Z-Score than patients treated with other antihypertensive drug classes or receiving no treatment in univariate and multivariate analysis (Table 4). The Z-Score for E/E’ in patients with RAS inhibition was 0.32 ± 0.64 vs 0.76 ± 1.18 without RAS inhibition (p = 0.02) in univariate analysis.

**Table 4:** Multivariable models of Tissue Doppler velocities, including E’/A’, E/E’ and S’.

	E’/A'		E/E’		S'	
	Estimate	P	Estimate	P	Estimate	P
Intercept	−0.28 ± 0.14	0.05	0.09 ± 0.50	0.86	−0.59 ± 0.39	0.14
Age (years)	−0.008 ± 0.008	0.27	0.04 ± 0.03	0.2	0.05 ± 0.02	0.03
Height Z-score	−0.03 ± 0.03	0.35	−0.26 ± 0.10	0.008	−0.001 ± 0.08	0.98
Male sex					0.57 ± 0.16	0.0009
eGFR	0.008 ± 0.002	0.002	−0.003 ± 0.008	0.71	−0.01 ± 0.006	0.07
Systolic BP Z-score	−0.07 ± 0.02	0.02				
ACEi/ARB therapy	0.13 ± 0.05	0.02	−0.37 ± 0.19	0.05		
E/E'	−0.05 ± 0.03	0.08			−0.40 ± 0.08	<0.0001
Concentric LV hypertrophy	0.08 ± 0.06	0.18			−0.25 ± 0.16	0.13

eGFR, estimated Glomerular Filtration rate (ml/min/1.73 m^2^); ACE, Angiotensine converting enzyme inhibitor; ARB, Angiotensine receptor blockade, E/E’ Early conventional to tissue Doppler diastolic velocity (cm/s); LV, left ventricular.

### Systolic function

Systolic tissue Doppler velocity Z-Scores were again mostly within normal ranges, however significantly reduced at the septal but not at the mitral annulus (Table 3, Fig. 1) compared to healthy controls. As described above, systolic function declined with increasing E/E’ Z-Scores. By multivariate analysis renal function and blood pressure were not associated with systolic tissue Doppler velocity Z-Scores, nor was any other recorded clinical or biochemical parameter predictive of reduced systolic function. Systolic function assessed by tissue Doppler Z-Scores was slightly lower in patients with concentric LVH in multivariate analysis. Systolic function assessed by midwall fractional shortening (MFS) and endocardial fractional shortening (EFS) were highly inter-correlated (r = 0.66, p < 0.0001, Table S2), MFS was positively correlated to E’ (r = 0.27, p < 0.05) and E’/A’ Z-Scores (r = 0.21, p < 0.05), no other tissue Doppler measurement was correlated to MFS or EFS.

### Left ventricular hypertrophy (LVH) and left ventricular geometry

Conventional echocardiographic analysis showed elevated LVMI and a high prevalence of LVH, which increased with declining renal function and was highest in dialysis patients (Table 2). Relative wall thickness was also increased, resulting in classification as concentric LV remodeling or concentric LV hypertrophy depending on LVMI in a substantial percentage of patients (Table 2).

LVMI increased with age, was negatively associated with height and higher in male patients. It was associated with systolic function expressed by midwall fractional shortening in univariate and multivariate analysis but not with Doppler velocity Z-Scores or systolic BP SDS (Tables S3 and S4). Concentric left ventricular remodeling and concentric LVH were associated with increased systolic BP SDS and reduced midwall fractional shortening but not with tissue Doppler velocities in multivariable logistic regression (Table 5). Left ventricular systolic tissue Doppler velocity Z-Scores showed a borderline negative association with concentric LV hypertrophy in multivariable analysis (Table 4).

**Table 5:** Multivariable logistic regression model for Concentric LVH and Concentric Remodeling

	Concentric LVH		Concentric Geometry	
	HR (95% CI)	p	HR (95% CI)	p
Age (years)	1.22 (1.05, 1.42)	0.011	0.991 (0.85, 1.15)	0.903
Height Z-score	0.65 (0.40, 1.07)	0.090	1.441 (0.84, 2.48)	0.185
Male sex	4.78 (1.36, 16.79)	0.015	1.37 (0.42, 4.44)	0.602
eGFR	1.00 (0.95, 1.04)	0.826	1.035 (0.99, 1.08)	0.149
Systolic BP Z-score	1.83 (1.17, 2.86)	0.008	1.61 (1.03, 2.52)	0.036
MFS	0.79 (0.68, 0.92)	0.002	0.59 (0.48, 0.73)	<0.0001

eGFR, estimated Glomerular Filtration rate (ml/min/1.73 m^2^; MFS, midwall fractional shortening; LVH, left ventricular hypertrophy.

## Discussion

This study in a large cohort of children and adolescents with CKD provides novel information about systolic and diastolic left ventricular function in this condition. Our study encompassed a comprehensive analysis of tissue Doppler derived indicators of diastolic function (early E’, late A’ wave, E’/A’, E/E’) and systolic function (S’). Measurements were normalized to age-independent z-scores^17^. We demonstrate a significant reduction of systolic and diastolic function in children with CKD compared to healthy children. A critical impact of renal function and blood pressure on diastolic function and a significant association of antihypertensive treatment with RAS blockade with improved left ventricular filling pressure (E/E’ Z-Score) and diastolic function (E’/A’ Z-Score) was evident. In turn, systolic function (S’ Z-Score) was positively influenced by reduced left ventricular filling pressure.

Smaller studies have obtained tissue Doppler measurements in children with CKD, but their interpretation of the results was usually based on small control cohorts of healthy children. Z-Scores based on published reference data have not been calculated. We therefore demonstrated that available normal data can and should be used to calculate valid Z-scores to interpret tissue Doppler measurements in children.

While there was a high prevalence of LVH typical of many CKD and dialysis cohorts^1,18,19^, there was no significant association of diastolic function assessed by tissue Doppler imaging to left ventricular mass or geometry and only a borderline association of systolic TD velocity (S’ Z-Score) to LV hypertrophy. Midwall fractional shortening was the only functional measure associated to concentric left ventricular morphology. Previous studies have linked E/E’ to left ventricular hypertrophy, but only if LVH was considered very pronounced (>97.5^th^ percentile)^18^ and in a smaller analysis unadjusted for age^20^. However, in the presence of LVH, diastolic or systolic dysfunction may play an additional predictive role for future adverse events^21^ or outcomes, as longitudinal analysis may reveal. More importantly, these results suggest that LV morphology alone does not sufficiently identify risk factors for cardiac involvement in children with CKD nor does it precisely enough describe cardiac alterations.

In previous small-sample studies in children with CKD variable Doppler indices such as tissue Doppler E’^16^, E’/A’^13,14^ or conventional pulse wave Doppler E/A^22^ were found to be reduced. While an association of diastolic function to serum Cystatin C levels has been shown^23^, no relationship to eGFR has been demonstrated. Our study, investigating a much larger patient cohort, is the first to show independent associations of both lower GFR and higher blood pressure with reduced diastolic function measured by TDI E’/A’ Z-Score. Additionally, diastolic function measured by E’/A’ Z-Score tended to be better preserved in patients treated with RAS blockade independently of renal function and blood pressure.

The Heart Failure and Echocardiography Associations of the European Society of Cardiology have recommended the evaluation of E/E’ in the workup of diastolic heart failure in adults^24^, however its role in childhood is not clear. Here, we observed a globally increased E/E’ Z-Scores, indicative of increased left ventricular filling pressure. In hypertensive adults and children with hypertrophic cardiomyopathy, E/E’ ratio emerged as a powerful predictor of cardiac events^21,25^. Also, the finding of the inverse correlation of E/E’ Z-Score with systolic TD velocitiy Z-Score may be of importance since our group previously described abnormal systolic mechanics in children with CKD similar to alterations in hypertrophic cardiomyopathy^5^.

We describe for the first time an independent association of RAS inhibition with better preservation of LV diastolic function in children with CKD. Notably, the association appeared to be specific for RAS blockers whereas the use of other antihypertensive agents, including diuretics, did not show any relationship with E/E’ Z-Score. Previous studies in pediatric CKD failed to show associations of LV filling pressure (E/E’) with antihypertensive treatment protocols, possibly due to insufficient sample size or lacking adjustment of Doppler measurements for patient age^14,16,22,26^. We therefore hypothesize that RAS blockade should be the preferred treatment for hypertension in children with CKD independent of the level of proteinuria.

Our study is also the first to demonstrate a close inverse relationship of E/E’ Z-Score with systolic function in children with CKD. This pathophysiological relationship emphasizes the importance of optimizing diastolic function, preferably by RAS inhibition although other aspects such as the fluid status of patients should not be neglected in order to improve diastolic function.

Furthermore, our results show a reduction not only of diastolic but also of systolic tissue Doppler velocities in children with CKD compared to healthy individuals, suggesting reduced systolic LV function. This finding confirms earlier findings obtained in pediatric CKD cohorts with various methodologies including analysis of fractional shortening^4^, TD^26^, and strain imaging^5^. Our analysis thus endorses the use of tissue Doppler imaging as a simple method to screen for subtle systolic dysfunction in the setting of normal left ventricular ejection.

While tissue Doppler imaging of the left ventricle is supposed to be more independent of preload than flow Doppler^27–29^, acute preload changes can impact also on tissue Doppler measurements^30^. In this study fluid status was not assessed directly. However, none of our patients was in a critically ill condition with acute volume changes, and patients on hemodialysis were examined between dialysis sessions in order to minimize the effects of acute volume changes. Future studies should however look more closely into the effect of fluid status on cardiac function in children with CKD.

Another obvious limitation of our study is the cross-sectional design of our analysis, which precludes any firm conclusions about causalities.

Chinappa *et al*. emphasized that the finding of subclinically decreased cardiac function in adults with CKD requires more research into functional mechanisms of cardiac disease in CKD^3^. Similarly, the Investigator Network Initiative Cardiovascular and Renal Clinical Trialists (INI-CRCT) has recently called for more trials evaluating the benefits and risks of cardiovascular medications in patients with CKD^31^. The observations made in this study underscore the presence of functional myocardial alterations and both the need and feasibility of using non-invasive imaging in interventional clinical trials in the pediatric subset of the CKD population.

In conclusion, we demonstrated that TD velocities are independent of LV mass and morphology and provide sensitive additional information about early alterations of the left ventricular function in children with CKD. These changes are independently related to the degree of renal functional impairment and systolic blood pressure. Treatment with RAS blockade may be beneficial not only for the prevention of CKD progression but also for cardiac sequelae.

## Methods

### Subjects and Study Design

The patients were participants of the 4C Study (The Cardiovascular Comorbidity in Children with Chronic Kidney Disease Study, registered at ClinicalTrials.gov NCT01046448^32^. The study prospectively observes 688 patients who were enrolled 2010–2012 at age 6 to 17 years with CKD stage III-V (eGFR < 60 ml/min/1.73 m^2^). The patients are followed prospectively with annual comprehensive cardiovascular assessments (including oscillometric BP measurements as previously described^2^ and echocardiographies) and 6-monthly clinical assessments, blood and urine collection. The measurement of Tissue doppler velocities was not part of the standard echocardiography protocol of the 4C study but was carried out as an ancillary measurement and therefore only available in a subset of patients who were seen by one investigator (A.D.). The patients were not selected specifically for this analysis but were included randomly due to their assignment to the performing investigator. The first available echocardiography per patient during the course of the study was included in the analysis.

The following Ethics committees (EC) and Institutional Review Boards (IRB) approved the 4C Study: Austria: EC of the University of Vienna; EC of the University of Innsbruck. Czech Republic: EC of the University of Prague. France: Comité de protection des personnes “Est IV”, Strasbourg. Germany: EC Charité— Universitätsmedizin Berlin; EC of the Medical Faculty of the University of Cologne; Ethikkommission der sächsischen Länderkammer; EC of Friedrich-Alexander-University Erlangen; EC of the University of Duisburg-Essen; EC of Albert-Ludwig University Freiburg; Ethikkommission der Ärztekammer Hamburg; EC and IRB of Hannover Medical School; EC and IRB of the University of Heidelberg; EC of the Medical Faculty of the University of Jena; EC of the Medical Faculty of the University of Leipzig; EC of Philipps University Marburg; Ethikkommission der Ärztekammer Westfalen-Lippe und der Medizinischen Fakultät der Westfälischen Wilhelms Universität Münster; EC of the Medical Faculty of the University of Rostock. Italy: Comitato Etico Indipendente dell’Azienda Ospedaliero-Universitaria di Bologna, Policlinico S. Orsola-Malpighi; Comitato di Etica dell’IRCCS Istituto Giannina Gaslini di Genova; Comitato Etico dell’IRCCS Ospedale Maggiore Policlinico, Mangiagalli e Regina Elena di Milano; Ethical Committee for clinical practice of the General Hospital-University of Padova; Comitato Etico per la Sperimentazione Clinica dell’IRCCS Ospedale Pediatrico Bambino Gesu’ di Roma. Lith- uania: EC and IRB of Vilnius University. Poland: EC at Children’s Memorial Health Institute, Warsaw. Portugal: EC of the University of Porto. Serbia: EC of the University of Belgrade. Switzerland: EC of the Canton Bern; EC of the Canton Zurich. Turkey: EC of the University of Cukurova, Adana; Non-interventional Clinical Researches Ethics Boards of Hacettepe Univer- sity, Ankara; EC of the University of Cerrahpasa, Istanbul; EC and IRB of the University of Ege, Izmir. United Kingdom: Great Ormond Street Hospital and UCL Institute of Child Health research EC. The study was performed in accordance with the ethical standards of the Declaration of Helsinki of 1964 and its later amendments. Written informed consent was given by the parents and adolescents, and oral assent by younger children.

For the present analysis 128 patients at 12 study sites underwent a standardized echocardiographic exam including tissue Doppler measurements at the time of the study visit. Clinical information and biospecimens collected at the same study visit were analyzed for the present study. GFR was calculated by the updated Schwartz formula^33^.

### Echocardiographic measurements

The transthoracic echocardiographies were performed according to the pediatric echocardiography standards issued by the American Society of Cardiology^34^. Patients on hemodialysis were examined between hemodialysis session in order to avoid acute volume changes. None of the patients was critically ill at the time of the investigation. The echocardiographic exam included standard M-mode images, pulsed Doppler and tissue Doppler echocardiography. The left ventricular mass index (LVMI) was calculated according to a recently published allometric formula providing age independent normalization of left ventricular mass (LVM/(height^2.16^ + 0.09)). An LVM index $\ge$ 45.0 m^2^ ^16^ was considered to identify LVH, representing the 95^th^ percentile^35^. Endocardial and midwall fractional shortening was calculated as suggested by Lang *et al*.^36^. Relative wall thickness was normalized to 10 years of age, using a cut-off of 0.38 as equivalent of the 95^th^ percentile^37^.

Tissue Doppler measurements included early (E’) and late (A’) diastolic and systolic (S’) velocity at the mitral and septal annulus of the left ventricle. Diastolic function was described by E’/A’ ratio and left ventricular filling pressure by E/E’ ratio.

All exams were performed by a single trained paediatrician (A.D.) and reviewed by an expert paediatric cardiologist (M.C.). All exams were performed on an Acuson P50, Siemens Healthcare, Erlangen. Image analysis was carried out using Syngo (Syngo US Workplace, Siemens Medical Solutions, USA Inc) as digital image evaluation software.

### Data analysis

Results of Tissue Doppler Imaging (TDI) measurements were normalized to patient age by calculation of Z scores based on a published reference cohort of 325 healthy children^17^. Standardized height and BMI were calculated from the WHO growth charts (http://www.who.int/growthref/en). Blood pressure values were standardized according to the Fourth Report on Diagnosis, Evaluation and Treatment of High Blood Pressure in Children and Adolescents^38^.

Data was tested for normal distribution by the Shapiro-Wilk test. Correlations of all variables of Table 1 to tissue Doppler measurements were tested with Spearman rank order correlation and group comparisons were carried out using chi-squared, t, or Mann–Whitney tests in case of two groups and chi- squared test, ANOVA, or Kruskal–Wallis test in case of more than two groups as appropriate. The influence of anthropometric, biochemical and disease–associated covariates as well as medication on tissue Doppler measurements and LV morphology was analyzed using multivariable linear, mixed and logistic regression modeling. Backward variable selection was performed with a threshold of P = 0.15. All multivariable models were controlled for the influence of age, height and eGFR.

## Supplementary information

Impaired Systolic and Diastolic Left Ventricular Function in Children with Chronic Kidney Disease - Results from the 4C Study, Supplemental Material

## Data Availability

The data that support the findings of this study are available from the corresponding author upon reasonable request.
