# Supplementary material for: Impaired Systolic and Diastolic Left Ventricular Function in Children with Chronic Kidney Disease - Results from the 4C Study
Source: Sci Rep. 2019 Aug 7;9:11462. doi: 10.1038/s41598-019-46653-3 (PMC6685994; doi:10.1038/s41598-019-46653-3)
Supplement: Supplementary file 1 — Impaired Systolic and Diastolic Left Ventricular Function in Children with Chronic Kidney Disease - Results from the 4C Study, Supplemental Material [file 41598_2019_46653_MOESM1_ESM.docx]

**Impaired Systolic and Diastolic Left Ventricular Function in Children with Chronic Kidney Disease - Results from the 4C Study, Supplemental Material**

Anke Doyon MD^1,18^, Pascal Haas MD^1^, Sevcan Erdem MD^2,18^, Bruno Ranchin MD^3,18^, Behrouz Kassai MD^4,^ Francesca Mencarelli MD^5,18^, Francesca Lugani MD^6,18^, Jerome Harambat MD^7,18^, Maria Chiara Matteucci MD^8,18^, Marcello Chinali^9,18^, Sandra Habbig MD^10,18^, Ariane Zaloszyc MD^11,18^, Sara Testa MD^12,18^, Enrico Vidal MD^13,18^, Charlotte Gimpel MB BChir MA^14,18^, Karolis Azukaitis^15,18^, Alexander Kovacevic MD^16^, Uwe Querfeld MD^17,18^, Franz Schaefer MD^1,18^

^1^ Division of Pediatric Nephrology, Center for Pediatrics and Adolescent Medicine, Heidelberg, Germany

^2^ Division of Pediatric Cardiology, Cukurova University, Adana, Turkey

^3^ Department of Pediatric Nephrology, Rheumatology and Dermatology, Hospices Civils de Lyon, France

^4^ Service de Pharmacotoxicologie, Centre d’Investigation Clinique, 1407 Inserm, UMR 5558, LBBE, CNRS Lyon, Université de Lyon and Hospices Civils de Lyon, France

^5^ Nephrology and Dialysis Unit, Department of Pediatrics, Azienda Ospedaliero Universitaria Sant Orsola-Malpighi, Bologna, Italy

^6^ Department of Pediatric Nephrology, Istituto Giannina Gaslini, Genova, Italy

^7^ Pediatric Nephrology Unit, Department of Pediatrics, Bordeaux University Hospital, France

^8^ Department of Nephrology and Urology, Bambino Gesù Pediatric Hospital, Rome, Italy

^9^ Department of Cardiology, Bambino Gesù Pediatric Hospital, Rome, Italy

^10^ University Childrens’ and Adolescents’ Hospital Cologne, Germany

^11^ Pediatric Nephrology Unit, Hautepierre University Hospital, Strasbourg, France

^12^ Pediatric Nephrology, Dialysis and Transplant Unit, Fondazione Osp Maggiore Policlinico, Milan, Italy

^13^ Pediatric Nephrology, Dialysis and Transplant Unit, Department of Womens and Childrens Health, University Hospital of Padova

^14^ Department of General Pediatrics, Adolescent Medicine and Neonatology, Center for Pediatrics, Medical Center – University of Freiburg, Faculty of Medicine, Germany

^15^ Center for Pediatrics, Vilnius University, Vilnius, Lithuania

^16^Department of Pediatric and Congenital Cardiology, Center for Pediatrics and Adolescent Medicine, Heidelberg, Germany

^17^ Division of Pediatric Nephrology, Charite Children’s Hospital, Berlin, Germany

^18^ 4C Study Consortium

**Running title:** Altered systolic and diastolic function in children of the 4C Study

**Correspondence address:**

Dr. Anke Doyon

Pediatric Nephrology Division

Center for Pediatrics and Adolescent Medicine

Im Neuenheimer Feld 430

69120 Heidelberg

Germany

Phone: 49 6221 56 37296

Fax: 49 6221 56 5166
Email: [anke.doyon@med.uni-heidelberg.de](mailto:anke.doyon@med.uni-heidelberg.deankeankank)

**Table S1.** Tissue Doppler Velocities in 128 children with CKD, stratified by CKD stage

|  | All | |  | CKD Stage | | |
| --- | --- | --- | --- | --- | --- | --- |
|  |  | |  | 2-3 | 4-5 | Dialysis |
| E’ |  | |  |  |  |  |
| Mitral anular | 18.5 ± 3.76 | |  | 18.9 ± 4.16 | 18.4 ± 3.54 | 17.6 ± 3.51 |
| Septal | 12.7 ± 2.32 | |  | 12.9 ± 2.38 | 12.6 ± 2.34 | 13.2 ± 2.11 |
|  |  | |  |  |  |  |
| A’ |  | |  |  |  |  |
| Mitral anular | 7.0 ± 1.92 | |  | 6.52 ± 1.64 | 6.98 ± 1.88 | 8.37 ± 2.47 |
| Septal | 6.39 ± 1.47 | |  | 6.10 ± 1.60 | 6.56 ± 1.40 | 6.51 ± 1.27 |
|  |  | |  |  |  |  |
| S’ |  | |  |  |  |  |
| Mitral anular | 10.8 ± 2.96 | |  | 10.6 ± 3.01 | 10.7 ± 2.95 | 11.7 ± 2.89 |
| Septal | 7.94 ± 1.39 | |  | 7.91 ± 1.26 | 7.85 ± 1.47 | 8.5 ± 1.31 |
|  |  | |  |  |  |  |
| E'/A' (diastolic function) | | |  |  |  |  |
| Mitral anular | 2.78 ± 0.67 | |  | 3.00 ± 0.67 | 2.75 ± 0.64 | 2.20 ± 0.42 |
| Septal | 2.08 ± 0.54 | |  | 2.22 ± 0.60 | 1.98 ± 0.49 | 2.09 ± 0.52 |
|  |  | |  |  |  |  |
| E/E' (Left ventricular compliance) | |  |  |  |  |  |
| Mitral anular | 5.45 ± 1.30 | |  | 5.26± 1.12 | 5.49 ± 1.37 | 5.95 ± 1.56 |
| Septal | 7.86 ± 1.74 | |  | 7.60 ± 1.58 | 8.08 ± 1.76 | 7.76 ± 2.20 |

*E’, Early diastolic TD velocity (cm/s); A’, Atrial TD velocity (cm/s); S’ Systolic TD velocity (cm/s)*

***Table S2:*** *Correlations of functional cardiac measures*

|  | ***E’*** | ***A’*** | ***S’*** | ***E’/A’*** | ***E/E'*** | ***MFS*** | ***EFS*** |
| --- | --- | --- | --- | --- | --- | --- | --- |
| ***E’*** | *-* | ***0.33‡*** | ***0.61‡*** | ***0.45‡*** | ***-0.50‡*** | ***0.27**** | *0.16* |
| ***A’*** | ***0.33‡*** | *-* | ***0.59‡*** | ***-0.59‡*** | ***-0.25†*** | *-0.03* | *0.02* |
| ***S’*** | ***0.61‡*** | ***0.59‡*** | *-* | *-0.06* | ***-0.41‡*** | *0.07* | *0.10* |
| ***E’/A’*** | ***0.45‡*** | ***-0.59‡*** | *-0.06* | *-* | ***-0.20**** | ***0.21**** | *0.10* |
| ***E/E'*** | ***-0.5‡*** | ***-0.25†*** | ***-0.41‡*** | ***-0.20**** | *-* | *-0.05* | *0.005* |
| ***MFS*** | ***0.27**** | *-0.03* | *0.07* | ***0.21**** | *-0.05* |  | ***0.66‡*** |
| ***EFS*** | *0.16* | *0.02* | *0.1* | *0.1* | *0.005* | ***0.66‡*** |  |

*E, Early conventional Doppler velocity; E’, Early diastolic tissue doppler velocity (cm/s); A’, Atrial tissue Doppler velocity (cm/s); S’ Systolic tissue doppler velocity (cm/s); MFS, midwall fractional shortening; EFS, endocardial fractional shortening*

*p<0.05 † p<0.001 ‡ p<0.0001

**Table S3:** Univariate correlation of LVMI and functional cardiac parameters

|  | **LVMI** |  |
| --- | --- | --- |
|  | Correlation coefficient | P |
| **E** | 0.10 | 0.328 |
| **E/A** | 0.06 | 0.570 |
| **E‘** | 0.011 | 0.905 |
| **A‘** | 0.083 | 0.350 |
| **E‘/A‘** | -0.05 | 0.591 |
| **E/E‘** | 0.07 | 0.513 |
| **S‘** | 0.08 | 0.393 |
| **EFS** | 0.04 | 0.66 |
| **MFS** | -0.20 | 0.034 |

*E, Early conventional Doppler velocity; E’, Early diastolic tissue Doppler velocity (cm/s); A’, Atrial tissue Doppler velocity (cm/s); S’ Systolic tissue Doppler velocity (cm/s); EFS, endocardial fractional shortening; MFS, midwall fractional shortening*

**Table S4:** Multivariable linear regression model of LVMI

|  | **LVMI** |  |
| --- | --- | --- |
|  | Estimate | P |
| Intercept | 45.6±6.19 | <.0001 |
| Age (years) | 0.46±0.27 | 0.09 |
| Height z-score | -1.81±0.94 | 0.06 |
| Male sex | 5.87±2.13 | 0.007 |
| eGFR | -0.16±0.08 | 0.06 |
| Systolic BP z-score | 0.71±0.81 | 0.38 |
| MFS | -0.52±0.26 | 0.05 |

*eGFR, estimated Glomerular Filtration rate (ml/min/1.73m^2^); MFS, midwall fractional shortening*
